# The Role of Plasma Interleukin-6 Levels on Atherosclerotic Cardiovascular Disease and Cardiovascular Mortality Risk Scores in Javanese Patients with Chronic Kidney Disease

**DOI:** 10.3390/jpm12071122

**Published:** 2022-07-10

**Authors:** Hendri Susilo, Mochammad Thaha, Budi Susetyo Pikir, Mochamad Yusuf Alsagaff, Satriyo Dwi Suryantoro, Citrawati Dyah Kencono Wungu, Nando Reza Pratama, Cennikon Pakpahan, Delvac Oceandy

**Affiliations:** 1 Doctoral Program of Medical Science, Faculty of Medicine, Universitas Airlangga, Surabaya 60132, Indonesia; hendrisusilo@staf.unair.ac.id; 2 Department of Cardiology and Vascular Medicine, Universitas Airlangga Hospital, Surabaya 60115, Indonesia; yusuf_505@fk.unair.ac.id; 3 Department of Internal Medicine, Faculty of Medicine, Universitas Airlangga, Surabaya 60132, Indonesia; 4 Department of Internal Medicine, Universitas Airlangga Hospital, Surabaya 60115, Indonesia; satriyo.dwi.suryantoro@fk.unair.ac.id; 5 Department of Cardiology and Vascular Medicine, Faculty of Medicine, Universitas Airlangga, Surabaya 60132, Indonesia; 6 Department of Physiology and Medical Biochemistry, Faculty of Medicine, Universitas Airlangga, Surabaya 60132, Indonesia; 7 Institute of Tropical Disease, Universitas Airlangga, Surabaya 60286, Indonesia; 8 Faculty of Medicine, Universitas Airlangga, Surabaya 60132, Indonesia; nando.reza.pratama-2016@fk.unair.ac.id; 9 Department of Biomedicine, Faculty of Medicine, Universitas Airlangga, Surabaya 60132, Indonesia; cennikon.pakpahan@fk.unair.ac.id; 10 Division of Cardiovascular Science, The University of Manchester, Manchester Academic Health Science Centre, Manchester M13 9PR, UK; delvac.oceandy@manchester.ac.uk

**Keywords:** chronic kidney disease, single nucleotide polymorphism, interleukin-6, cardiovascular disease

## Abstract

Interleukin-6 (IL-6) has been identified as an important pro-inflammatory factor involved in mediating the severity of chronic kidney disease (CKD). This study sought to determine the effect of plasma IL-6 levels on atherosclerotic cardiovascular disease (ASCVD) and cardiovascular mortality risk scores in Javanese CKD patients. We also analyzed the frequency of IL-6 G174C single nucleotide polymorphism (SNP) in the population. This study was a cross-sectional study involving seventy-three patients of Javanese ethnic origin with stable chronic kidney disease. We assessed the ASCVD risk score, cardiovascular mortality score, genotyping of IL-6 G174C SNP, and plasma IL-6 levels in these patients. The genotype distribution and allele frequencies of the IL-6 G174C SNP were predominated by the G genotype/allele (GG: 97.26%, GC: 1.37%, CC: 1.37%, G-allele: 97.95%, and C-allele: 2.05%). Despite the fact that plasma IL-6 levels did not directly affect cardiovascular mortality risk, further analysis revealed its direct effect on the ASCVD risk score (path coefficient = 0.184, *p* = 0.043, 95% CI = 0.018–0.380), which in turn affected cardiovascular mortality risk (path coefficient = 0.851, *p* = <0.01, 95% CI = 0.714–0.925). In conclusion, plasma IL-6 levels play important roles on ASCVD risk and cardiovascular mortality risk in Javanese patients with CKD.

## 1. Introduction

The Kidney Disease Improving Global Outcomes (KDIGO) defines chronic kidney disease (CKD) as an abnormality in kidney structure or function that lasts for more than three months [1,2]. The prevalence of CKD is estimated to be 13.4% (11.7–15.1%) worldwide. Patients with end-stage kidney disease (ESKD) who require renal replacement treatment are estimated to be between 4.902 and 7.083 million worldwide [3], showing that the burden of CKD occurs in almost all countries [4]. In Asian countries, CKD prevalence was 9.8% (8.3–11.5%) in upper-middle-income countries and 13.8% (9.9–18.3%) in lower-middle-income countries [5]. In Indonesia, until 2017, as many as 27,232,922 CKD cases resulted in deaths. Cardiovascular complications are still among the most common causes of death in CKD [6]. Interactions between the heart and kidney intersect at multiple levels. The failing heart could hemodynamically cross-talk with the kidneys, and vice versa, in which CKD can occur due to complications from diabetes and hypertension, while cardiovascular disease is the most common complication and cause of death in CKD [7,8].

The inflammatory process plays a significant role in the progression of CKD to mortality. One of the proinflammatory cytokines that play an essential role in the pathogenesis of CKD is interleukin-6 (IL-6) [9]. IL-6 release is stimulated by acute infection, chronic inflammatory conditions, obesity, and physiological stress [10]. IL-6 is also associated with atherosclerosis and cardiovascular disease, which may also be a vital mediator of the inflammatory response in ischemic stroke [11]. Blood vessels are responsive to IL-6 generated from vascular and non-vascular sources. IL-6 signaling mediates various effects on blood vessel walls, including endothelial activation, vascular permeability, immune cell recruitment, endothelial dysfunction, and vascular hypertrophy and fibrosis [12].

Genetic involvement in the IL-6 response has been investigated in several studies [13,14]. The human IL-6 gene is located on chromosome 7p21, consisted of five exons and four introns, and synthesized as a 232-amino acid precursor protein [15]. IL-6 functional promoter single nucleotide polymorphism (SNP), G174C (rs1800795), has been identified previously and may be associated with elevated IL-6 levels [15,16]. Elevated IL-6 levels are related to higher mortality rate in people with cardiovascular diseases [17]. Proinflammatory cytokines upregulate the expression of matrix metalloproteinases, which are involved in vascular remodeling and plaque disruption. The presence of an inflammatory process marks the location of plaque rupture or erosion [18]. The possible impact is the progression of CKD, leading to severe cardiovascular complications [19,20].

However, some studies regarding IL-6 G174C polymorphism still yield conflicting results. The GG genotype has been linked to various ischemic and atherosclerotic cardiovascular diseases [21,22,23]. Other research, on the other hand, have discovered links between the CC genotype and asymptomatic carotid artery atherosclerosis and higher mortality in individuals with abdominal aortic aneurysms [24,25]. Some research even showed no significant difference in the allelic or genotype frequency between cardiovascular disease and control [26,27]. These contradicting results are likely due to differences among studies, including variations in ethnicity, study design, baseline characteristics, and population background [28,29,30].

To date, there has been no data on the effect of IL-6 polymorphisms and IL-6 levels on the risk of atherosclerotic cardiovascular disease (ASCVD) and cardiovascular mortality in CKD patients, especially in Javanese ethnicity, the largest ethnic group in Indonesia. Therefore, we conducted a study to determine the role of IL-6 G174C polymorphism and plasma IL-6 levels on ASCVD and cardiovascular mortality risk scores in Javanese CKD patients.

## 2. Materials and Methods

### 2.1. Study Design

This was an observational analytical study with a cross-sectional design to analyze the role of IL-6 G174C gene polymorphism and IL-6 plasma levels with atherosclerotic cardiovascular disease and cardiovascular mortality risk scores in Javanese CKD patients. The study is a continuation of our previous study focusing on the effect of polymorphism in the ACE gene on atherosclerotic cardiovascular disease and cardiovascular mortality risk [31]. We have added several CKD patients to be included in the present study. In brief, 73 CKD patients between May 2021 to June 2021 in the Nephrology Outpatient Clinic, Universitas Airlangga Hospital, Surabaya, Indonesia, were included in this study. This study has been approved by the Institutional Ethics Committee of Universitas Airlangga Hospital (ethical clearance number 146/KEP/2021).

### 2.2. Sample Criteria

The inclusion criteria were described in our previous paper [31]. Briefly, samples included in this study should fulfill the following inclusion criteria: (1) aged 40–79 years; (2) clinically stable CKD patients; and (3) Javanese ethnicity. Clinically stable CKD was defined as not in conditions of metabolic acidosis, hyperkalemia, overload syndrome, and severe infection [32]. Patients with present cardiovascular disease or past cardiovascular histories such as acute coronary syndrome, stroke, acute heart failure, severe infection, and uncontrolled arrhythmias were excluded. A physical and history examination were carried out before taking blood for DNA isolation. Retrieved baseline characteristics included gender, age, ethnicity, body mass index (BMI), blood pressure, history of smoking, diabetes, stroke, and CKD stage. We calculated the ASCVD risk score for all patients for measuring the 10-year estimate of atherosclerotic cardiovascular disease. We also calculated the cardiovascular mortality score for predicting 10-year cardiovascular mortality in the patients. Both calculations were based on the SCORE CKD patch (https://ckdpcrisk.org/ckdpatchscore/, accessed on 10 November 2021) and the PCE CKD patch (https://ckdpcrisk.org/ckdpatchpce/, accessed on 10 November 2021) [33]. Peripheral blood (5 mL) was collected from all patients in EDTA tubes. The blood was taken to the Institute of Tropical Diseases (ITD), Universitas Airlangga, Indonesia. The salting-out method was employed to extract peripheral blood mononuclear cells (PBMC) from the samples. Blood plasma from each sample was also isolated and put into an Eppendorf tube. PBMC and blood plasma were stored in −80 °C for at least one month before further processing.

### 2.3. Plasma IL-6 Levels

Enzyme-linked immunoassay (ELISA) kit (Cat. No. E-EL-H6156, Elabscience, Houston, TX, USA) was used to measure IL-6 levels in the plasma as specified by the manufacturer’s procedure. The outcomes would then be read in ELISA HumaReader and analyzed in ELISA for Windows software (Center for Disease Control and Prevention, https://www.cdc.gov/ncird/software/elisa/index.html, accessed on 23 August 2021).

### 2.4. DNA Isolation and Genotyping of IL-6 Gene SNP

DNA was isolated with the QIAamp DNA extraction kit (Qiagen, Inc., Hilden, Germany; Cat. No. 51104) as instructed in the reaction kit. To detect the IL-6 G174C polymorphism (rs1800795), we used the polymerase chain reaction-restriction fragment length polymorphisms (PCR-RFLP) method. Each amplification reaction used Promega GoTaq^®^ Green Master Mix (Cat. no. M7122, Promega, Madison, WI, USA) in A DNA thermal cycler machine (Applied Biosystems Veriti 96 Well). The primers used were: Forward: 5′-TGACTTCAGCTTTACTCTTTG-3′, Reverse: 5′-CTGATTGGAAACCTTATTAAG-3′ (198 bp amplicon). The procedure includes initial denaturation at 95 °C–5′, 40 cycles: 95 °C–30″, 52 °C–30″, 72 °C–45″, and final extension at 72 °C–5′. PCR products were then incubated with restriction enzyme NIaIII (Cat. No. R0125S, New England Biolabs, Ipswich, MA, USA) at 37 °C for 4 h [34]. The results of the PCR-RFLP showed 119 and 49 bp bands for minor homozygote (SNP) CC genotype, 30 and 168 bp for major homozygote GG genotype, and three bands (168, 119, and 49 bp) for heterozygote CG genotype. We also performed direct sequencing to confirm the results of the PCR-RFLP.

### 2.5. Data Analysis

SPSS Statistics Software version 23 was used for statistical analysis (IBM Corp., Armonk, NY, USA). The baseline characteristics and IL-6 genotype distributions were presented in tabular form and analyzed descriptively. Numerical data were evaluated using mean and standard deviation (SD), while the frequency was calculated using percentage. Shapiro–Wilk normality test was employed to determine the distribution of the numerical data. To determine the relationship between variables in this study with ASCVD and cardiovascular mortality scores, Spearman analysis was used. Smart PLS 3.3.7 (GmbH Company, Oststeinbek, Germany) was used to determine the path analysis between the plasma IL-6 level, ASCVD risk score, and cardiovascular mortality risk score. Statistical significance was defined as a *p*-value less than 0.05.

## 3. Results

### 3.1. Characteristics of the Participants

**Figure 1:**
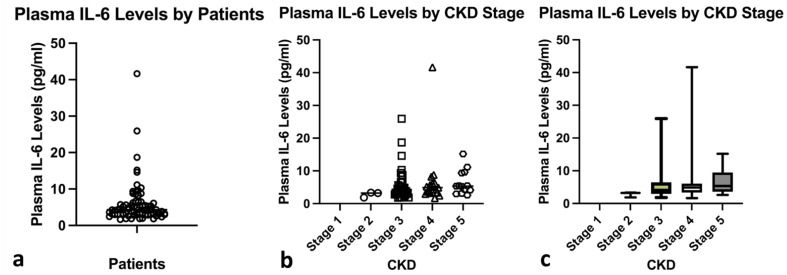
The level of plasma IL-6 in: (**a**) total patients; (**b**) patients were stratified according to CKD stages; (**c**) box-plot of plasma IL-6 levels based on CKD stages.

Of the total seventy-three Javanese CKD patients involved in this study, the mean age was 57.93 ± 7.15 years with male predominance (52.1%). The medical history regarding cardiovascular risk factors revealed that 76.7% of the CKD patients had diabetes, 87.7% had hypertension, and 69.9% never smoked (Table 1). Most of the patients had stage 3 CKD (52.1%) with high ASCVD risk score (23.83 ± 19.82) and cardiovascular mortality risk score (17.06 ± 19.45). The total mean of plasma IL-6 was 5.92 ± 5.83 pg/mL, while there was no difference in plasma IL-6 levels between CKD stages (Figure 1). We also performed additional analysis to determine the correlation between IL-6 levels and eGFR, however, we found no statistically significant correlations (*p* = 0.164). Correlation between plasma IL-6 levels and CKD stages was also not statistically significant (*p* = 0.054). In Mann Whitney test, we found statistically significant differences between male and female in terms of ASCVD risk and cardiovascular mortality scores (*p* = 0.001 and *p* = 0.001, respectively).

### 3.2. Correlation and Path Analysis between Plasma IL-6 Level, ASCVD Risk Score, and Cardiovascular Mortality Risk Score

**Figure 2:**
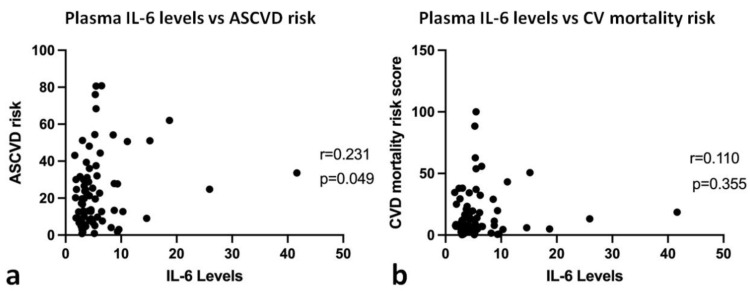
XY correlation plot between plasma IL-6 and ASCVD and CV mortality risk scores: (**a**) XY correlation plot between plasma IL-6 and ASCVD risk score; (**b**) XY correlation plot between plasma IL-6 and CV mortality risk score. Plasma IL-6 levels were measured at pg/mL unit.

**Figure 3:**
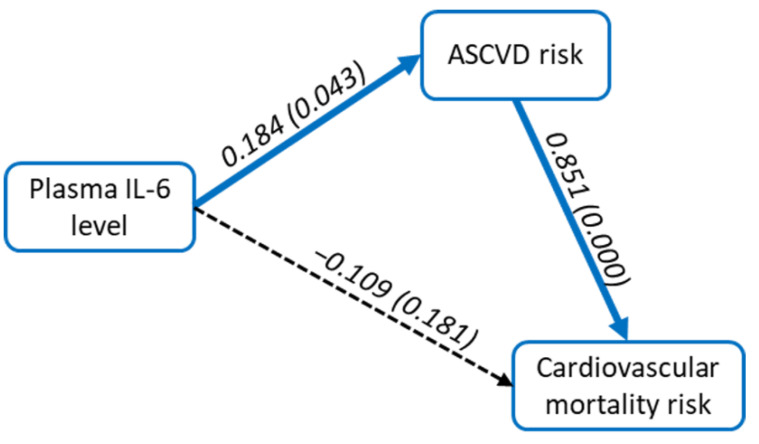
Path analysis between plasma IL-6 level, ASCVD risk score, and cardiovascular mortality risk score.

In the present study, we found a positive correlation between plasma IL-6 and ASCVD risk score (r = 0.231, *p* = 0.049). Still, there was a not significant correlation between plasma IL-6 and cardiovascular mortality risk score (r = 0.110, *p* = 0.355), as shown in Figure 2. We also performed path analysis to determine the relationship between plasma IL-6 level, ASCVD risk score, and cardiovascular mortality risk score (Figure 3). We found that plasma IL-6 level had a significant direct effect on ASCVD risk (path coefficient = 0.184, *p* = 0.043, 95% CI = 0.018–0.380), while ASCVD risk score had a significant direct effect on cardiovascular mortality risk score (path coefficient = 0.851, *p* = <0.01, 95% CI = 0.714–0.925). Plasma IL-6 level had a significant indirect effect on cardiovascular mortality risk score (path coefficient = 0.156, *p* = 0.045, 95% CI = 0.016–0.344). Plasma IL-6 level did not directly affect cardiovascular mortality risk (*p* = 0.181), implying that there could be several other factors influencing ASCVD risk to affect cardiovascular mortality risk.

### 3.3. Analysis of IL-6 G174C SNP in Javanese CKD Patients

**Figure 4:**
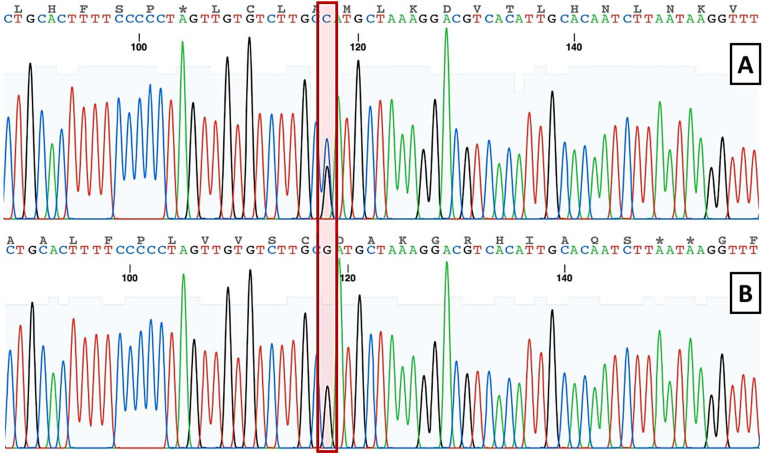
Identification and genotyping of SNP using direct sequencing method for confirmation: (**A**) chromatogram of GC genotype (heterozygote); (**B**) chromatogram of GG genotype (major homozygote).

**Figure 5:**
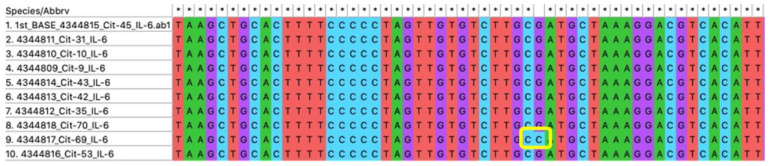
Multiple alignments of IL-6 gene to detect G174C polymorphism by direct sequencing.

It has been known that plasma IL-6 level is significantly correlated with the G174C polymorphism [19,25,36]. To detect the frequency of IL-6 G174C SNP in this study participants, we performed PCR-RFLP followed by direct sequencing for confirmation. Chromatogram comparison and multiple alignments between SNP and major homozygote are presented in Figure 4 and Figure 5. As shown in Table 2, in this study, we only found one Javanese CKD patient with GC genotype (1.37%) and one patient with CC genotype (1.37%). According to the allele calculation, only three C alleles were found in this study (2.05%). The frequency of the G allele (100%) and C allele (0%) in this study population seemed to be comparable with the reported frequency in the Asian population (dbSNP, NCBI) [37]. As the number of polymorphic alleles in this cohort was very low (only one subject each in both GC and CC genotypes), we did not perform correlation analysis due to the limited power of the statistical test.

## 4. Discussion

Atherosclerosis is an inflammatory disease characterized by a chronic inflammatory process perpetuated by various proinflammatory mediators, including cytokines and chemokines, at all stages of the disease, with myocardial infarction, stroke, or sudden cardiac death as fatal endpoints [38]. Among other cytokines, IL-6 is considered an orchestrator of the inflammatory response and a key player in atherosclerosis in humans [10]. IL-6 has been shown to play an essential role in atherogenesis by inducing endothelial dysfunction, enhanced expression of adhesion molecules, the proliferation of smooth muscle cells, leukocyte recruitment, and matrix degeneration [39,40,41]. Studies demonstrated that IL-6 could also promote the development and rupture of atherosclerotic plaques, thereby accelerating the progress of atherosclerotic plaque growth and instability [42]. As chronic inflammation is pervasive at all CKD stages, IL-6 levels are elevated in CKD [19]. However, this phenomenon is partially explained by reduced renal clearance of this cytokine [43]. Furthermore, previous research has shown that high IL-6 had been solidly associated with mortality in patients with stage five CKD who are being maintained on long-term dialysis [44,45,46]. Non-traditional risk factors such as oxidative stress and inflammation have an important role in increasing the progression of cardiovascular disease in CKD, as there is a bidirectional relationship between oxidative stress and inflammation. The increase in markers of oxidative stress in CKD has started to occur since the early stage of CKD [47,48].

The present study results corroborated previous findings on the link between increased serum IL-6 and the risk of cardiovascular diseases [49,50]. Several previous studies have probed the association between serum IL-6 and cardiovascular mortality with conflicting results. A prior study among women with prevalent CVD demonstrated that those with higher plasma IL-6 levels had a more than the fourfold risk of death than women in the lowest tercile [18]. Tuomisto et al. on the other hand, found that CRP and TNF-α, but not IL-6, were significant independent predictors of total mortality in men [51]. However, another cohort study found IL-6 more strongly associated with all-cause and cardiovascular mortality than CRP [52]. The small sample size may explain these conflicting findings in several studies, the participants’ old age, and heterogeneous populations. However, in the present study among relatively homogenous populations, we found that plasma IL-6 levels did not directly affect cardiovascular mortality risk score, implying that there could be several other factors influencing ASCVD risk that can affect cardiovascular mortality risk. Gender difference could also be an important covariate. We found in this study that there were significant differences between male and female CKD patients in terms of ASCVD risk and cardiovascular mortality risk scores. Between males and women, there are significant disparities in ASCVD risk. Men are more likely to acquire coronary heart disease (CHD) and often develop ASCVD at a younger age [53,54]. It is unclear why sex-based disparities could influence ASCVD risk and mortality. However, it has frequently been hypothesized that sex steroid hormones, notably estrogen, reduce the incidence of ASCVD in women [55]. This hypothesis was strongly supported by several studies which showed that estrogen therapy proves to effectively reduce total mortality and coronary heart disease in postmenopausal women [56,57].

The proinflammatory cytokine IL-6 has a great many functions, including stimulating the hepatic synthesis of acute-phase reactants, activating endothelial cells, increasing coagulation, and promoting lymphocyte proliferation and differentiation [39]. The different effects acting on the various stages of CAD would influence the development, progression, and complications of the disease. Several possible key mechanisms are thought to be involved in the development and progression of CAD by IL-6. First, serum IL-6 is the main stimulator of the hepatic acute-phase response, which is associated with increased blood viscosity and increased platelet number and activity [58]. Second, the autocrine and paracrine activation of monocytes by IL-6 in the vessel wall contributes to the deposition of fibrinogen [59]. Acute-phase response proteins such as CRP and fibrinogen are both strong risk factors for CAD. Third, IL-6 makes the low-density lipoprotein receptor (LDLR) show up on the surface of macrophages. This makes macrophages more likely to take in low-density lipoprotein (LDL), which speeds up lipid deposition and promotes foam cell formation [60]. Fourth, circulating IL-6 also stimulates the hypothalamic-pituitary-adrenal (HPA) axis, the activation of which is associated with central obesity, hypertension, and insulin resistance [61]. The above findings suggest that IL-6 plays a vital role in the pathology of atherosclerosis and contributes to the development of CAD through multiple pathways.

Identifying and analyzing polymorphisms in genes encoding biochemical markers that are altered in patients with CKD is critical as they may affect the patients’ outcomes [62]. In this study among Javanese CKD patients, we found that the frequency of IL-6 G174C SNP was very low; thus, we could not analyze any associations between IL-6 G174C SNP and plasma IL-6 level, ASCVD risk score, or cardiovascular mortality risk score. Several studies have suggested that promoter polymorphisms might affect the transcriptional regulation of IL-6. However, studies on the link between the IL-6 G174C SNP and ASCVD and cardiovascular mortality risk have shown conflicting results. Prior research conducted in Germany [25], Italy [19], and South Africa [36] established that the IL-6 G174C SNP was associated with increased circulating levels of IL-6, as well as an increased risk of developing cardiovascular disease and mortality in CKD patients. According to the findings of a meta-analysis of 42 studies involving 15,145 cases and 21,496 controls, the C allele of the IL-6 G174C SNP was associated with an increased risk of cardiovascular disease in Caucasians [63]. Additionally, other studies discovered a link between the CC genotype and the C allele of the IL-6 G174C SNP and the onset of cardiovascular events, establishing it as a risk factor for myocardial infarction [64,65]. In contrast, previous research has shown that the IL-6 G174C SNP is not associated with an increased risk of cardiovascular disease in Tunisians [66], Chinese [67], or the Isfahan population [68].

It is well established that the frequency of various cytokine gene alleles varies between populations. In our study, the genotype distribution and allele frequencies of the IL-6 G174C SNP were dominated by the G allele (GG: 97.26%, GC: 1.37%, CC: 1.37%; G-allele: 97.95%, C-allele: 2.05%). According to the Reference SNP (rs) Report database for rs1800795, which was accessed on 23 March 2022 [37], the allelic frequencies in our studied population are closer to those reported for Asian populations. Similar findings have been made in populations of Africans, East Asians, and Malaysian Malays [28,69,70]. Our findings also backed up an earlier study in Indonesia that looked at different ethnic groups and found that the GG genotype is the most common, while the CC genotype is less common [71]. In comparison, prior research among the European Caucasian population discovered that the GC genotype is the most prevalent [72,73]. Thereby, when considering the effect of the IL-6 G174C SNP, it is necessary to consider the influence of population genetics, particularly in complex and multifactorial diseases such as ASCVD and CKD.

This study is the first in our knowledge to explore the relationship between IL-6 G174C SNP, IL-6 levels, ASCVD risk score, and cardiovascular mortality risk score in Javanese CKD patients. However, this study has some limitations that should be considered when assessing the data’s relevance. Although the research was performed in a relatively homogeneous population, the sample size was relatively small. Notably, we only assessed one SNP locus (G174C) SNP genotype, while the role of other IL-6 promoter SNPs remains to be elucidated. To our knowledge, there was a dearth of data on the genetic regulation of IL-6 in patients with chronic kidney disease in our population. Nonetheless, this study is representative of Javanese CKD populations, and it supported the validity of previous findings.

## 5. Conclusions

In conclusion, our data indicate that plasma IL-6 levels play a significant role on ASCVD risk and cardiovascular mortality risk scores in CKD patients. We also found that the GG genotype of the IL-6 G174C SNP was predominant in the majority of subjects in Javanese populations. Based on the insights gained from this study, further research on other IL-6 loci or even haplotype studies are needed to elaborate on these findings, particularly among Asian populations.

## Figures and Tables

**Table 1:** Characteristic of the Javanese CKD patients in this study.

Variable	Value (*n* = 73)
Gender, male (%)	38 (52.1)
Age (years)	57.93 ± 7.15
History of type 2 diabetes (%)	56 (76.7)
History of hypertension (%)	64 (87.7)
History of smoking	
Non-smoker *n* (%)	51 (69.9)
Current smoker *n* (%)	4 (5.5)
Former smoker *n* (%)	18 (24.7)
Stages of kidney disease	
CKD stage 2 *n* (%)	3 (4.1)
CKD stage 3 *n* (%)	38 (52.1)
CKD stage 4 *n* (%)	20 (27.4)
CKD stage 5 *n* (%)	12 (16.4)
Dyslipidemia *n* (%)	58 (79.5%)
BMI (Kg/m^2^)	26.08 ± 5.16
SBP (mmHg)	144.27 ± 23.13
DBP (mmHg)	81.29 ± 11.92
Total cholesterol (mg/dL)	183.96 ± 52.22
HDL (mg/dL)	39.75 ± 12.35
Serum creatinine (mg/dL)	2.64 ± 1.66
e-GFR (mL/min/1.73 m^2^)	31.82 ± 14.94
Urine ACR (mg/gram)	643.33 ± 973.73
Plasma IL-6 (pg/mL)	5.92 ± 5.83
ASCVD risk score (%)	23.83 ± 19.82
Cardiovascular mortality risk score (%)	17.06 ± 19.45

BMI: Body mass index; SBP: Systolic blood pressure; DBP: Diastolic blood pressure; HDL: High-density lipoprotein; e-GFR: estimated glomerular filtration rate; ACR: albumin-creatinine ratio; ASCVD: atherosclerotic cardiovascular disease. Dyslipidemia was defined as total cholesterol >200 mg/dL or HDL cholesterol <40 mg/dL [35].

**Table 2:** Distribution of IL-6 genotypes in this study.

Genotype	*n*	Frequency (%)	IL-6 Plasma (pg/mL)	ASCVD Risk Score (%)	Cardiovascular Mortality Risk Score (%)	CKD Stage (%)
GG	71	97.26	5.48 ± 4.04	23.90 ± 19.99	17.18 ± 19.74	Stage 2 (4.2)
						Stage 3 (52.1)
						Stage 4 (26.8)
						Stage 5 (16.9)
GC	1	1.37	1.874	9.30	7	Stage 3 (100)
CC	1	1.37	41.66	33.60	18.50	Stage 4 (100)
Total	73	100				
**Allele**	***n***	**Frequency (%)**				
G	143	97.95				
C	3	2.05				
Total	146	100				

## Data Availability

All relevant data are within the paper.
